# Frontotemporal craniotomy with orbital osteotomy for superior hypophyseal artery aneurysm clipping

**DOI:** 10.3171/2023.10.FOCVID23157

**Published:** 2024-01-01

**Authors:** Grant Arzumanov, Seung W. Jeong, Bhavika Gupta, Rocco Dabecco, Jose Sandoval, Richard Williamson, Alexander Yu

**Affiliations:** Department of Neurosurgery, Allegheny General Hospital, Pittsburgh, Pennsylvania

**Keywords:** exoscope, superior hypophyseal aneurysm, microscopic clipping, improved ergonomics

## Abstract

Superior hypophyseal artery (SHA) aneurysms are rare paraclinoid aneurysms with a mortality rate as high as 3%–6%. Surgical clipping of these aneurysms is technically challenging due to the surrounding anatomy. The large size and complicated surrounding anatomy make endovascular coiling very difficult. Here we present the case of a ruptured right SHA aneurysm. The authors present technical nuances of the clipping using an exoscope rather than a traditional microscope.

The video can be found here: https://stream.cadmore.media/r10.3171/2023.10.FOCVID23157

## Transcript

This video highlights the benefits of the use of exoscope visualization in anterior skull base surgery with a clipping of a ruptured superior hypophyseal artery region aneurysm. Benefits of exoscope visualization include increased magnification using optical and digital zoom, improved surgeon ergonomics, and improved visualization angles. When comparing the traditional microscope and exoscope, surgeon ergonomics are improved as the visualization device is no longer attached to the surgeon. Due to the independence of the visualization device from the surgeon, a wide range of operative angles are possible while maintaining the ergonomics of the surgeon. In this example, we demonstrate the ability to visualize and perform appropriate microdissection work without changing patient positioning and while maintaining surgeon ergonomics.

### 1:15 Exoscope Setup.

In this picture the surgeon is at the top of the patient’s head toward the right side. The exoscope Modus X (Synaptive) faces behind the surgeon, and the exoscope arm reaches over the surgeon’s head. Multiple 3D viewing devices are available, with the main screen directly opposite the surgeon. With this setup the surgeon moves around the operative field while maintaining the appropriate visualization of the exoscopic field.

### 1:39 Clinical Presentation and Neurological Exam.

This is a 33-year-old male presenting with the worst headache of his life. He was intact on examination and was diagnosed with a Hunt-Hess grade 2, Fisher 3 subarachnoid hemorrhage.

### 1:52 Preoperative Neuroimaging Findings.

CTA of the head demonstrates a medially pointing aneurysm on the right proximal supraclinoid internal carotid artery. There was a concern for a contralateral aneurysm; however, the angiogram did not demonstrate this. Digital subtraction angiography demonstrates a partially thrombosed aneurysm in the region of the superior hypophyseal artery.

### 2:16 Rationale for Surgery.

Rationale of the procedure was to secure the aneurysm and prevent rerupture.^1,2^ Risks of the procedure include, but are not exclusive to, hemorrhage, infection, stroke, coma, and death. Benefits include the potential cure for the aneurysm and prevention of rerupture. Discussions of observation, endovascular strategies, and microsurgical treatment were made.^3^ Given his age, partial thrombosis of the aneurysm, and the wide neck nature of the aneurysm, clip ligation was felt to be most appropriate.^4^

### 2:57 Surgical Setup.

The patient was placed in three-point fixation in a supine position with the head turned toward the aneurysm’s contralateral side with the malar eminence at the top. A one-piece fronto-temporo-orbital craniotomy with orbital osteotomy was utilized for improved visualization.

Extradural clinoidectomy with sectioning of the falciform ligament was performed to improve safe mobilization of not only the aneurysm but also the optic nerve.^1,2,5^ This was also performed for improved proximal control in case of intraoperative rupture. The cervical carotid artery was also visible. SSEPs and MEPs were utilized. Burst suppression was utilized upon dissection of the neck of the aneurysm until the aneurysm was secure. ICG was available but not utilized as Doppler was deemed sufficient.

### 3:45 Surgical Procedure.

Once the bone flap has been elevated, the optic nerve is identified by the ovoid shape of bone overlying the entering nerve. Orbital roof is removed in piecemeal up to the optic canal. Transition from optical to digital zoom is seamless as noted here. A high-speed diamond drill bit is used with Kobe’s irrigation to eggshell the optic canal. The anterior clinoid process is drilled down. Remaining anterior clinoid process is removed in piecemeal, and with curette. The thin remainder of bone overlying the optic canal is then removed. The dura is then opened in a C-shaped fashion. The arachnoid overlying the optic nerve is released. The falciform ligament is then elevated and cut, further relaxing the optic nerve. The medial carotid is released. The posterior communicating artery is identified. The distal and proximal neck of the aneurysm is then identified.^5^

### 5:32 Clipping of the Aneurysm.

Initially, a right-angled fenestrated clip was planned, but given the intraoperative visualization of a wider neck, the aneurysm was subsequently clipped for carotid reconstruction with the initial clip being placed on the presumed rupture point, the lateral portion of the aneurysm. Another right-angle clip is placed on the medial aspect of the carotid. The tines can be seen overlapping the other clip completing the obliteration of the aneurysm. Another clip was placed to obliterate a small dog-ear.

### 6:33 Postoperative Course.

Patient did well and was at neurological baseline postoperatively. No visual issues on examination or subjectively, relating to clip interference of the optic nerve, intraoperative manipulation, nor injury to the superior hypophyseal or carotid artery perforators supplying the optic nerve. Patient did not have any hormonal complaints or deficits relating to pituitary dysfunction. Postoperative CTA and digital subtraction angiography of the head showed complete obliteration of the aneurysm.

### 7:17 Conclusions.

This case demonstrates the benefits of exoscopic visualization. Benefits include increased magnification using optical and digital zoom. Surgeon ergonomics are improved, as the visualization device is no longer attached to the surgeon. The patient position was not changed during the intracranial portion of the procedure, and the surgeon was able to visualize the anatomy to perform the surgery safely and effectively while maintaining good ergonomics.

## Disclosures

Dr. Williamson reported personal fees from Synaptive Medical during the conduct of the study.

## Author Contributions

Primary surgeon: Yu. Assistant surgeon: Arzumanov, Jeong, Sandoval. Editing and drafting the video and abstract: Arzumanov, Jeong, Gupta, Dabecco, Sandoval. Critically revising the work: all authors. Reviewed submitted version of the work: Arzumanov, Jeong, Dabecco, Sandoval, Williamson. Approved the final version of the work on behalf of all authors: Yu. Supervision: Yu, Arzumanov, Dabecco, Williamson.

## Supplemental Information

### Patient Informed Consent

The necessary patient informed consent was obtained in this study.
